# Commentaries on “Associations of Lower Extremity Muscle Strength, Area, and Specific Force With Lower Urinary Tract Symptoms in Older Men: The Baltimore Longitudinal Study of Aging”

**DOI:** 10.1093/gerona/glae102

**Published:** 2024-05-17

**Authors:** Yu-Hsiang Lin, Han-Yu Tsai, Yu-Ting Chen

**Affiliations:** Department of Geriatric Urology, Chang Gung Memorial Hospital-Linkou, KweiShan, TaoYuan, Taiwan; School of Medicine, Chang Gung University, KweiShan, TaoYuan, Taiwan; Department of Geriatric Urology, Chang Gung Memorial Hospital-Linkou, KweiShan, TaoYuan, Taiwan; School of Medicine, Chang Gung University, KweiShan, TaoYuan, Taiwan; Department of Geriatric Urology, Chang Gung Memorial Hospital-Linkou, KweiShan, TaoYuan, Taiwan; School of Medicine, Chang Gung University, KweiShan, TaoYuan, Taiwan

**Figure 1.**
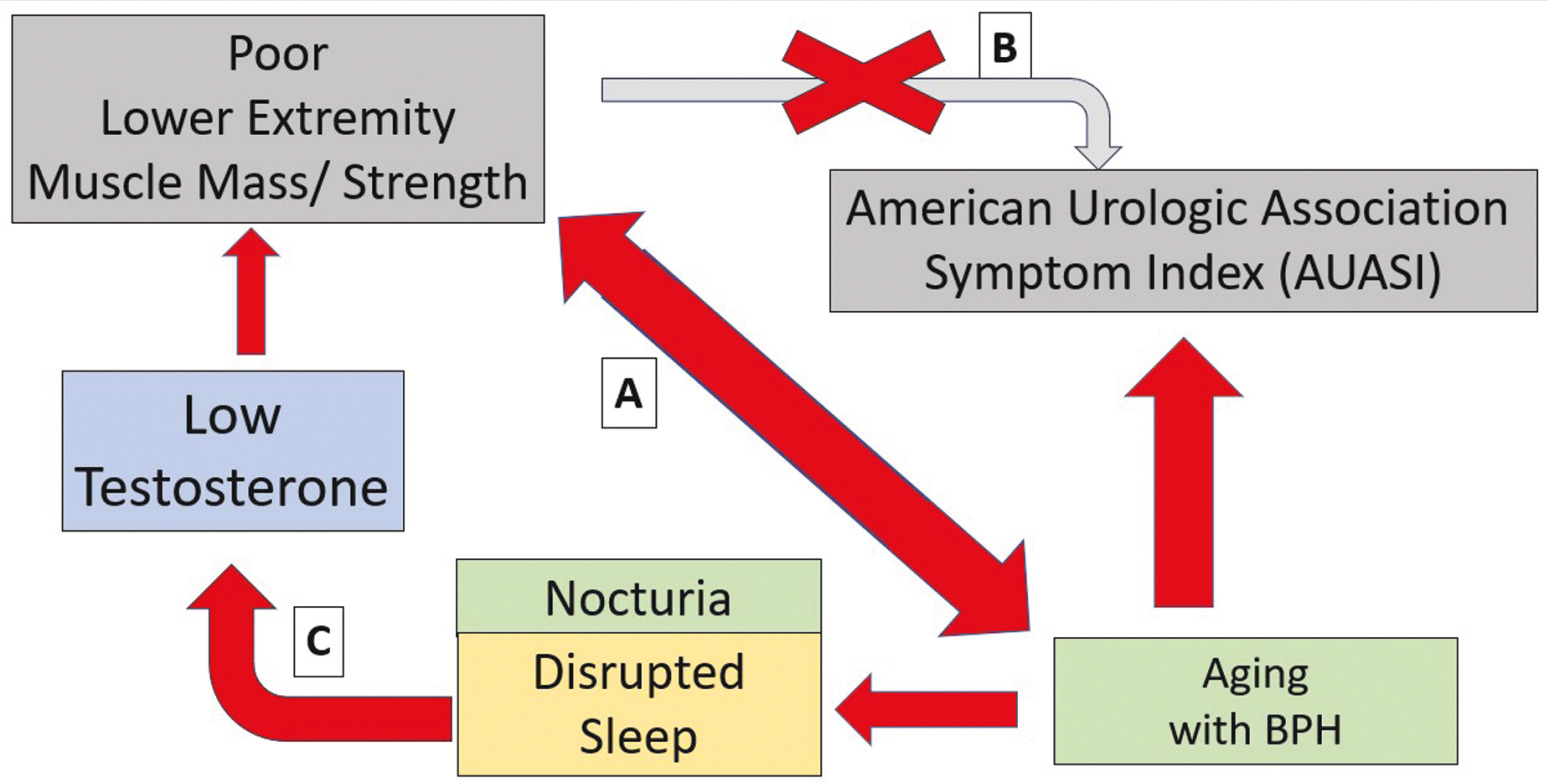
Interconnected pathways between aging, nocturia, testosterone levels, and muscle strength in older men with benign prostatic hyperplasia (BPH). This figure illustrates the theorized interactions among aging with BPH, nocturia with disrupted sleep, and their subsequent effects on testosterone levels and lower extremity muscle mass/strength in older men. Path A indicates a significant association between lower extremity muscle strength and BPH, supported by cross-sectional data showing that higher muscle strength and specific force correlate with milder lower urinary tract symptoms (LUTS). Path B denotes the absence of a longitudinal relationship between baseline muscle strength and the progression of LUTS as measured by the American Urologic Association Symptom Index (AUASI). Path C suggests a potential feedback loop where nocturia, through disrupted sleep, may lead to decreased testosterone levels, which could in turn lead to a decline in muscle mass and strength.

After a thorough reading of the study by Langston et al. (1), we were compelled to reflect on the intricate relationship between lower extremity muscle strength, area, specific force, and lower urinary tract symptoms (LUTS) in older men. This comprehensive analysis, utilizing the Baltimore Longitudinal Study of Aging to assess 352 men aged 60 and above, highlighted a correlation in cross-sectional models between higher thigh muscle strength and specific force with milder LUTS. However, it’s the longitudinal analyses that sparked our curiosity, as they failed to reveal a significant connection between baseline lower limb muscle measurements and annual changes in urinary symptoms, suggesting a nuanced complexity in the evaluation of skeletal muscle function in LUTS assessments for older men. (See Figure 1 for a schematic representation of the hypothesized relationships.)

As the prevalence of sarcopenia increases with age, leading to a notable reduction in muscle mass and strength (2,3), and given the concurrent exacerbation of prostate enlargement and the acceleration of urinary symptoms in men (4), one might intuitively predict a correlation similar to that proposed by Langston et al. Contrary to expectations, the longitudinal analyses do not support a significant relationship between muscle metrics and urinary symptom progression. This outcome led us to speculate that the causal link might be inverted: As urinary symptoms worsen, they could, in turn, contribute to the decline in lower limb muscle power. This hypothesis introduces testosterone as a potential intermediary in this relationship, considering its association with muscle mass, strength, and the aggravation of nocturia due to sleep disruption (5–7).

Further exploration into the complex interplay of benign prostatic hyperplasia (BPH) and nocturia by Lin et al. (8) elucidates how BPH-induced nocturia suppresses antidiuretic hormone (ADH), thereby increasing total nighttime urine volume and contributing to a decrease in testosterone levels. Bladder outlet obstruction, a fundamental issue in men with BPH, leads to increased bladder pressure. This pressure triggers a cascade of pathological changes, including bladder remodeling and fibrosis, which consequently reduce bladder capacity. The diminished capacity of the bladder results in a noticeable uptick in the frequency of urination, especially problematic during the night. Nighttime urination interrupts sleep, affecting the hypothalamus’s circadian rhythm control center. A significant outcome of this disruption is the reduced secretion of ADH, which aggravates nocturnal polyuria by diminishing the kidneys’ ability to concentrate urine. The amalgamation of decreased bladder capacity and increased urine volume causes frequent nocturnal awakenings (nocturia), thus perpetuating the cycle of reduced ADH secretion. This cycle exacerbates the severity of nocturia.

Moreover, sleep disturbances due to nocturia extend their disruptive influence beyond ADH regulation, affecting the hypothalamus–pituitary–gonad (HPG) axis. This disruption dampens testosterone secretion, essential for maintaining muscle strength and mass. Reduced testosterone levels exacerbate BPH (9) and adversely affect muscle health, leading to diminished muscle strength and area. Therefore, nocturia not only instigates a hormonal imbalance cycle affecting ADH but also triggers another harmful cycle by affecting the HPG axis and lowering testosterone levels. These interconnected cycles highlight the intricate relationship between BPH-induced alterations, hormonal regulation, and muscle health, emphasizing the challenge of breaking these cycles without targeted intervention.

Several studies have shown that the severity of the American Urologic Association Symptom Index for assessing LUTS does not correlate with testosterone levels (10,11). This introduces a compelling proposition: By revisiting the causality between baseline muscle strength and LUTS progression with a focus on nocturia or testosterone levels as analytical parameters, a significant connection might be uncovered between nocturia or testosterone and lower extremity muscle strength, area, and specific force.

Building on this insight, we believe that managing nocturia is paramount, as it is the catalyst for the self-reinforcing vicious cycles involving ADH and the HPG axis’s testosterone. Preventing the accelerated decline of testosterone is crucial for maintaining muscle mass and strength, a hypothesis that necessitates further observation and experimentation to validate.
